# Impact of diversity of *Mycoplasma hyopneumoniae* strains on lung lesions in slaughter pigs

**DOI:** 10.1186/s13567-016-0408-z

**Published:** 2017-01-17

**Authors:** Annelies Michiels, Katleen Vranckx, Sofie Piepers, Rubén Del Pozo Sacristán, Ioannis Arsenakis, Filip Boyen, Freddy Haesebrouck, Dominiek Maes

**Affiliations:** 1 Department of Reproduction, Obstetrics and Herd Health, Unit Porcine Health Management, Faculty of Veterinary Medicine, Ghent University, Salisburylaan 133, 9820 Merelbeke, Belgium; 2 Applied Maths, Sint-Martens-Latem, Belgium; 3 Department of Pathology, Bacteriology and Avian Diseases, Faculty of Veterinary Medicine, Ghent University, Salisburylaan 133, 9820 Merelbeke, Belgium

## Abstract

The importance of diversity of *Mycoplasma hyopneumoniae* (*M. hyopneumoniae*) strains is not yet fully known. This study investigated the genetic diversity of *M. hyopneumoniae* strains in ten pig herds, and assessed associations between the presence of different strains of *M. hyopneumoniae* and lung lesions at slaughter. Within each herd, three batches of slaughter pigs were investigated. At slaughter, from each batch, 20 post mortem bronchoalveolar lavage fluid samples were collected for multiple locus variable-number tandem repeat analysis (MLVA), and lung lesions (*Mycoplasma*-like lesions, fissures) were examined. Multivariable analyses including potential risk factors for respiratory disease were performed to assess associations between the number of different strains per batch (three categories: one strain, two–six strains, ≥seven strains), and the lung lesions as outcome variables. In total, 135 different *M. hyopneumoniae* strains were found. The mean (min.–max.) number of different strains per batch were 7 (1–13). Batches with two–six strains or more than six strains had more severe *Mycoplasma*-like lesions (*P* = 0.064 and *P* = 0.012, respectively), a higher prevalence of pneumonia [odds ratio (OR): 1.30, *P* = 0.33 and OR: 2.08, *P* = 0.012, respectively], and fissures (OR = 1.35, *P* = 0.094 and OR = 1.70, *P* = 0.007, respectively) compared to batches with only one strain. In conclusion, many different *M. hyopneumoniae* strains were found, and batches of slaughter pigs with different *M. hyopneumoniae* strains had a higher prevalence and severity of *Mycoplasma*-like lung lesions at slaughter, implying that reducing the number of different strains may lead to less lung lesions at slaughter and better respiratory health of the pigs.

## Introduction

*Mycoplasma hyopneumoniae* (*M. hyopneumoniae*) is the causative agent of enzootic pneumonia, and infections occur in all countries with an intensive pig production [1]. Infections with *M. hyopneumoniae* cause tremendous economic losses, either directly or indirectly, by increasing the susceptibility of infected animals to other respiratory pathogens [2].

*Mycoplasmas* have small genomes (580–1300 kb) [3, 4], and genetic diversity might be one solution to adapt to the adverse environment of the host [5, 6]. Many regions in the genome of *M. hyopneumoniae* related to adherence in the host contain variable number of tandem repeats (VNTRs). These regions are prone to recombination events and slipped strand mispairing, which can possibly lead to expression of a different sized protein [7]. Multiple locus variable number of tandem repeat analysis (MLVA) has been used successfully to genetically characterize *M. hyopneumoniae* isolates [8–12]. This technique has a high discriminatory power, and can be applied directly to clinical samples without the necessity to grow the bacterium, which is very fastidious in the case of *M. hyopneumoniae* [9].

Previous studies have shown that there is a high diversity of *M. hyopneumoniae* field isolates, especially between strains from different herds [10]. Other studies including a limited number of herds not practising vaccination against *M. hyopneumoniae*, showed that in some herds, only one strain was detected, whereas different strains were found in other herds, even in the same pig [9, 12]. The importance of genetic diversity of *M. hyopneumoniae* strains however is not fully known. A possible link between the presence of multiple simultaneous or subsequent infections with different *M. hyopneumoniae* strains and the presence and severity of lung lesions has been suggested [9, 10, 13], but no systematic study has been conducted to answer this question. If the presence of different *M. hyopneumoniae* strains is associated with more clinical disease and/or lung lesions, then measures decreasing the diversity of strains may be helpful to control respiratory problems in pig herds.

The aim of this study was to investigate the presence of different *M. hyopneumoniae* strains in consecutive batches of slaughter pigs from different herds, to type the strains using MLVA and to investigate associations between the occurrence of multiple strains of *M. hyopneumoniae* and the prevalence and severity of lung lesions.

## Materials and methods

### Study population

A list of herds (*n* = 56) complying with following criteria: closed herd or closed production system, herd with at least 100 breeding sows and vaccination of piglets against *M. hyopneumoniae* was provided by one of the largest slaughter houses in Belgium (Covalis). The list of these farms was randomized (Excel 2010, Microsoft Corp., Redmond, WA, USA) and the farmers were contacted in order of appearance on the randomized list until ten herds willing to participate to the study were obtained. Descriptive data of the ten study herds are presented in Table 1. Different potential risk factors for respiratory disease were collected from these herds during a herd visit by the first author. During the visit, a questionnaire was completed, the stables were visited and the fattening pigs inspected. The potential risk factors in the questionnaire were based on previous studies [14] and pertained to biosecurity, management, housing and vaccination status (Table 2).

**Table 1:** **Description of the ten study herds (A–J) enrolled in the study**

Herd	A	B	C	D	E	F	G	H	I	J
Number of sows	170	200	250	200	150	250	200	250	150	125
Sows breed	LW (50%) + ELR (25%) + FLR (25%)	Topigs	ELR (50%) + FLR (50%)	Danbred (70%) + Hypor	Topigs	Topigs	Danbred	Rattlerow-Seghers	Hypor (90%) + Danbred (10%)	Hypor
Batch production system for sows	3-week	3-week	4-week	3-week	Day system	1-week	4-week	1-week	4-week	4-week
Stocking density nursery (m^2^/animal)	>0.30	<0.30	<0.30	>0.30	<0.30	>0.30	<0.30	>0.30	>0.30	<0.30
Stocking density fatteners (m^2^/animal)	>0.70	>0.7	0.65–0.70	0.65–0.7	0.7	<0.65	0.65–0.70	0.65–0.70	0.65–0.70	0.65
Purchase of gilts (occasions per year)	No	Yes (5)	No	Yes (4)	Yes (4)	No	No	Yes (8)	Yes every month^a^	Yes (5)
Duration of quarantine period for gilts	n.a.	9 week	n.a.	8 week	4 week	n.a.	n.a.	No	4 week	6 week
*Mycoplasma hyopneumoniae* vaccination gilts	No	Yes	No	Yes	No	No	No	No	Yes	No
Age (days) at vaccination of piglets against *M. hyopneumoniae*	8 and 26	21	14	7	14	7	35	3–8 and 28	18	21
Other vaccinations in piglets	No	PCV-2 (21)	No	PRRSv (18)	PCV-2 (14)	No	PCV-2 (35)	No	PCV-2 (18)	No
Age at weaning (days of age)	26	26	21	23	25	24	19	28	20–21	21
Clinical signs of *M. hyopneumoniae*	No	No	No	Yes	Yes	No	No	No	No	No
Coughing score for fattening pigs provided by the farmer (0–10)	3	2	0	7	4	2	3	0	1	0

LW: large white, ELR: English land race, FLR: French land race, n.a.: not applicable, PCV-2: porcine circovirus type 2, PRRSv: porcine reproductive and respiratory syndrome virus.

^a^Schedule of purchasing gilts has been accelerated with transition to Danbred.

**Table 2:** **Potential risk factors for respiratory disease that were collected from the ten herds**

Continuous variables	
Times per year farmer is purchasing gilts	“X” times per year that the farmer purchased gilts
Number of herds surrounding the herd in a perimeter of <5 km	calculated with Lambert coordinates and the Pythagoras theorem
Number of sows present on the herd	Measure for the size of the herd
Production system for the sows	0: no week system, 1, 2, 3, 4-week system
Coughing score given by the farmer	(0–10) fatteners
Categorical variables	
Purchase of gilts	1 = yes, 0 = no
Purchase of gilts always from the same supplier	1 = yes, 0 = no
Quarantine period for gilts	1 = yes, 0 = no
Herd located close to a highway (<5 km)	1 = yes, 0 = no
Herd located near a slaughter house (<5 km)	1 = yes, 0 = no
Distance herd to the public road (<100 or >100 m)	1 (<100 m), 2 (>100 m)
Sow breed	0: Topigs, 1: LW + ELR + FLR, ELR + FLR, Danbred + hypor, Danbred, RA-SE, Hypor
Dynamic or stable groups for pregnant sows	Stable (0) or dynamic (1) group sows
AIAO farrowing unit	1 = yes, 0 = no
AIAO nursery unit	1 = yes, 0 = no
AIAO fattening unit	1 = yes, 0 = no
Stocking density nursery	1 < 0.30 m^2^/pig; 2 > or = 0.30 m^2^/pig
Cross fostering during first week of life piglets	0 = no, 1 < 10%, 2 ≥10%
Cross fostering after first week of life piglets	0 = no, 1 < 10%, 2 ≥ 10%
Stocking density fattening unit	1 ≥ 0.70 m^2^/pig, 2 = 0.70–0.65 m^2^/pig, 3 < 0.65 m^2^/pig
Cleaning and disinfection farrowing unit	1 = yes, 0 = no
Cleaning and disinfection nursery	1 = yes, 0 = no
Cleaning and disinfection fattening unit	1 = yes, 0 = no, 2 = only cleaned not disinfected
Stand empty period farrowing unit	1 = yes, 2 = not always, 0 = no
Stand empty period nursery unit	1 = yes, 2 = not always, 0 = no
Stand empty period fattening unit	1 = yes, 2 = not always, 0 = no
Gilts vaccinated against *M. hyopneumoniae*	1 = yes, 0 = no
Clinical signs of *M. hyopneumoniae* in grower-finishers	1 = yes, 0 = no

LW + ELR + FLR: large white, English landrace, French landrace, ELR + FLR: English landrace, French landrace, RA-SE: Rattlerow-Seghers.

### Sampling at the slaughterhouse and lung lesion scoring

Three different batches of fattening pigs per herd were evaluated at the slaughterhouse during a time span of one to three months. All visits were performed from November 2012 until April 2013. From each batch, 20 randomly selected blood samples were collected at exsanguination, and from 20 other randomly selected pigs, the lungs were collected. For practical reasons, only the left half of the lung was taken. The blood samples and lungs were transported to the laboratory of Bacteriology of the Faculty of Veterinary Medicine, Ghent University immediately after the slaughterhouse visit.

Additionally, as many lungs as possible of each batch (min. 50) were evaluated for lung lesions. The lungs that were sampled were not included in the lung lesion scoring. The lungs were scored for presence of pneumonia and severity of *Mycoplasma*-like lesions using the method described by Morrison et al. [15]. *Mycoplasma*-like lesions were defined as macroscopic greyish to purplish consolidated pneumonia areas, generally located on the cranio-ventral parts of the lung lobes. The lungs were also evaluated for the presence of fissures and pleurisy. Fissures were defined as areas of collapsed alveoli adjoining alveolar emphysema (recovery lesions) [16], while pleurisy was defined as fibrotic adherences between the parietal and visceral membranes of the pleural cavity [17]. No approval of the ethical committee of Ghent University was necessary, as the pigs were destined for slaughter.

### Nested polymerase chain reaction (NPCR)

Upon arrival in the laboratory, the lung halves were flushed with 20 mL phosphate buffered saline (PBS, 8 g/L NaCl, 0.34 g/L KH_2_PO4, 1.21 g/L K_2_HPO_4_, pH 7.3). The recovered fluid was centrifuged at 2000* g* during 30 min to obtain the remaining pellet by carefully removing the supernatant. The pellet was resuspended in 1 mL of PBS and 200 µL of the resuspension was used to perform the DNA extraction using the DNeasy blood and tissue kit (Qiagen, Belgium) according to the instructions in the protocol manual. *Mycoplasma hyopneumoniae*-DNA was detected with a two steps nested polymerase chain reaction (nPCR) [18]. The nPCR products were analyzed by gel electrophoresis on a 1.5% agarose gel in Tris–Borate–EDTA (TBE)-buffer and stained with GelRed™ (Biotium. Inc., CA, USA) with visualization under UV illumination.

### Multiple locus variable-number tandem repeat analysis (MLVA)

All nPCR positive samples were submitted to a multiplex PCR as previously described [9]. Briefly, loci h1, h5 repeat 2, p97 repeat 1 and p146 repeat 3 were amplified in a multiplex reaction with a mastercycler epgradient S (Eppendorf, Hamburg, Germany) in a final volume of 20 µL containing 1× PCR buffer [20 mM Tris–HCl (pH 8.4), 50 mM KCl], 3 mM MgCl_2_, 0.2 mM deoxynucleotide triphosphate, 0.75 U of Platinum^®^ *Taq* DNA Polymerase (Invitrogen, Merelbeke, Belgium), 0.1 μM of each primer and finally 2 µL of template DNA. Ten cycles (30″94 °C; 30″63 °C; 1′15″69 °C) in which the annealing temperature was incrementally decreased with 1 °C per cycle were performed. Next, forty cycles (30″94 °C; 30″53 °C; 1′15″69 °C) and a final extension step of 5 min at 69 °C followed.

The PCR-products were diluted 1:10 with high performance liquid chromatography filtered water (HPLC–H_2_O). Amplicons were kept at 4 °C for a maximum of 48 h. A volume of 165 µL Hi-Di formamide (one run, 16 samples) (Applied Biosystems, Halle, Belgium) or a multitude of 165 µL for multiple runs was pipetted in an 1.5 µL Eppendorf (Eppendorf Belgium N.V.-S.A, Rotselaar, Belgium) and 1.5 µL of 600 LIZ standard (Applied Biosystems, Halle, Belgium) was added. 10 µL of this mixture was added to 1 µL of sample (PCR-product). Samples were denatured at 95 °C for 5 min, cooled on ice and electrophoresis was applied on the ABI 3130xl genetic analyzer (Applied Biosystems) for 16 samples at 15 kV during 14 000 s at 65 °C or for more than 16 samples on the ABI 3730xl (Applied Biosystems) at 15 kV during 14 000 s at 70 °C.

The resulting electropherogram files were imported into BioNumerics version 7.5 (Applied Maths, Sint-Martens-Latem, Belgium). After normalization, the VNTR numbers were calculated automatically from the detected peaks. A minimal spanning tree was constructed with the Prims’ algorithm using the multistate categorical coefficient. Only samples for which all four loci were detected, were included in the tree. A weight factor was assigned to each locus according to its’ allelic variation in the obtained dataset with the highest weight assigned to the locus with the lowest variation. Following weights were assigned to each locus: 2, 3, 3 and 6 to p146, h1, h5, and p97, respectively. A strain was defined as a unique MLVA-type, e.g. if the combination of repeat numbers was unique. Clonal complexes were defined when strains differed in no more than one locus, with the exception of the most stable locus p97. The Hunter-Gaston discriminatory index was calculated for the complete dataset, as well as for each herd [19].

### Serology

The sera of the blood samples (20 per batch) were tested for presence of antibodies against *M. hyopneumoniae* using a blocking ELISA (IDEIA™ *Mycoplasma hyopneumoniae* EIA kit, Oxoid Limited, Hampshire, UK). Sera with optical density (OD) <50% of the average value of the OD-buffercontrol were considered to be positive (ELISA *M. hyopneumoniae* positive samples). All values above or equal to 65% of the average value of the OD-buffercontrol were classified as negative. All doubtful samples equal to 50% and less than 65% of the average value of the OD-buffercontrol were considered to be negative as well.

Eight of the 20 samples from each batch were also tested for presence of antibodies against porcine reproductive and respiratory syndrome virus (PRRSv) (HerdCheck PRRS X3, IDEXX, Liebefeld-Bern, Switzerland) and subtypes H1N1, H1N2 and H3N2 of swine influenza virus (SIV) (standard haemagglutination-inhibition test).

### Statistical analyses

Different statistical models were used to assess the associations between the number of strains on the one hand and the presence and severity of lung lesions on the other hand. The number of different strains found in each batch of pigs was categorized as follows: category 1 (CAT 1): one *M. hyopneumoniae* strain per batch, category 2 (CAT 2): two to six different strains per batch, and category 3 (CAT 3): ≥seven different strains per batch. The category one strain per batch was used as reference; the classification in category 2 and 3 was made to obtain the same number of strains in these categories.

The number of strains per batch was considered as explanatory variable in the models. As lung lesions may not only be caused by infection with *M. hyopneumoniae* and/or determined by the number of strains, the effect of the different potential risk factors for respiratory disease (Table 2) was also taken into account in the models. A forward selection procedure was used during the model building, and risk factors with a *P* value >0.15 were removed. Remaining risk factors (with *P* value <0.15) were tested for collinearity. Correlations were assessed using Pearson’s (continuous variables) or Spearman rank (categorical variables) correlation, and in case two variables were highly correlated (∣r∣ > 0.6), the most significant factor was retained. In the final model, only risk factors with a *P* value <0.05 were retained. Confounding factors were identified when the regression coefficient (β) of another risk factor deviated more than 25% or 0.1 when β < 0.4 when removing the factor from the model. Such factors were excluded, but mentioned below each model. In total, four separate multivariable models were tested. The outcome variables for the different models were: severity of *Mycoplasma*-like lesions, likelihood of pneumonia lesions, fissures and pleurisy. Ln-transformation of the severity of the *Mycoplasma*-like lesions was performed to normalize the data. In all models, herd and lung were included as a random effect and batch was included as fixed effect.

A linear mixed regression model (MLwiN 2.26 [20]) was used to assess the influence of category of number of strains on the severity of the *Mycoplasma*-like lesions in each batch. The assumptions of normality and homogeneity of variance of the final model were tested by examining normal probability plots of residuals and plots of residuals versus predicted values. No patterns indicating heteroscedasticity were present. The multilevel linear regression model may be represented mathematically as: Y_ij_ = β_0_ + β_1_category 2_ij_ + β_2_category 3_ij_ + batch2_ij_ + batch3_ij_ + ε_ij_, where Y_ij_ is the continuous outcome variable (severity of *Mycoplasma*-like lesions), βs are the model coefficients, category is the fixed effect of the category of different number of strains, batch is the fixed effect of batch 1–3, herd is the random effect of herd *i* (*i* = 1–10), j refers to the jth lung in the ith herd and ε_ij_ is the random error term, assumed to be normally distributed with mean 0 and variance σ^2^.

Logistic mixed regression models using 1st order marginal quasi-likelihood algorithms were used to assess the influence of strain category on the likelihood of pneumonia, fissures and pleurisy (MLwiN 2.26—Centre for Multilevel Modeling, Bristol, UK [20]). The fit of the models was evaluated by inspection of the lung standardized residuals plotted against the normal scores and the lung level predicted values. The Hosmer–Lemeshow goodness-of-fit measure was calculated for the explanatory variable models using SAS 9.3 (PROC LOGISTIC, SAS Institute Inc., NC, USA). The results were represented as odds ratio (OR) with the 95% confidence interval calculated around these odds ratios. The multilevel logistic regression model may be represented mathematically as: g(Y_ij_) = β_0_ + β_1_category 2_ij_ + β_2_category 3_ij_ + batch2_ij_ + batch3_ij_ + ε_ij_, where (g) refers to the logit link function, Y_ij_ is the probability of the outcome variable on the logit scale (likelihood of pneumonia, fissures and pleurisy), β_s_ are the model coefficients, category is the fixed effect of category of number of strains, batch is the fixed effect of batch 1–3, herd is the random effect of herd *i* (*i* = 1 to 10), j refers to the jth lung in the ith herd and ε_ij_ is the random error term, assumed to be normally distributed with mean 0 and variance σ^2^.

## Results

### Descriptive results of the nPCR, MLVA, lung lesions and serology

#### Nested PCR

From the 600 bronchoalveolar fluid samples, 495 (82.5%) tested positive using nPCR for *M. hyopneumoniae*. The average percentage of positive samples in each category (Table 3) per batch were: CAT 1: 42.5%, CAT 2: 79.6% and CAT 3: 91.1%. In all batches of each herd, nPCR positive samples were detected. The descriptive nPCR results for each herd and for each batch per herd separately are shown in Table 4.

**Table 3:** **Descriptive results in the three category groups:** prevalence of nPCR positive results, average number of different strains, severity of *Mycoplasma*-like lesions ±SD, prevalence pneumonia, fissures and pleurisy expressed in percentages

	Category			Overall
	1	2	3	
nPCR results	42.5	79.6	91.1	82.5
Average number of different strains	1	4	9	7
Severity of *Mycoplasma*-like lesions ± SD	0.78 ± 2.4	3.97 ± 10.7	5.54 ± 12.7	4.59 ± 11.7
Prevalence of pneumonia	11.8	23.2	29.7	25.9
Prevalence of fissures	29.2	41.4	42.3	41.3
Prevalence of pleurisy	10.2	21.2	29.1	24.6

Severity of *Mycoplasma*-like lesions: minimum 0% and maximum 100% of the lung surface affected with pneumonia.

1 = batches with only one strain detected, 2 = batches with 2–6 different strain and 3 =  batches with ≥7 strains detected, SD: standard deviation, *n*:  number, nPCR results: nested polymerase chain reaction: percentage of positive animals for *M. hyopneumoniae*-DNA detected in the bronchoalveolar lavage fluid.

**Table 4:** **Descriptive results of the strain data of ten herds and the three batches (1–3) within each herd:** prevalence of nPCR (*n* = 600) positive results, number of different *M. hyopneumoniae* strains, total number of *M. hyopneumoniae* strains and number of bronchoalveolar lavage fluid samples obtained with detection of double or triple different strains

Herd	A	B	C	D	E	F	G	H	I	J	Total
nPCR	88 (53/60)	63 (38/60)	87 (52/60)	80 (48/60)	95 (57/60)	83 (50/60)	98 (59/60)	100 (60/60)	63 (38/60)	66 (40/60)	83 (495/600)
1	100 (20/20)	55 (11/20)	85 (17/20)	95 (19/20)	100 (20/20)	95 (19/20)	95 (19/20)	100 (20/20)	15 (3/20)	5 (1/20)	75 (149/200)
2	65 (13/20)	55 (11/20)	90 (18/20)	90 (18/20)	85 (17/20)	90 (18/20)	100 (20/20)	100 (20/20)	90 (18/20)	95 (19/20)	86 (172/200)
3	100 (20/20)	80 (16/20)	85 (17/20)	55 (11/20)	100 (20/20)	65 (13/20)	100 (20/20)	100 (20/20)	85 (17/20)	100 (19/20)	87 (174/200)
Number of different strains	16	6	10	19	18	23	14	15	12	7	135
1	9	2	5	8	11	13	6	10	2	1	65
2	9	3	6	9	8	11	9	6	8	4	71
3	7	1	3	6	10	7	4	5	3	3	49
Number of strains	67	11	66	49	94	46	69	53	23	18	496
1	25	5	15	18	32	17	21	20	2	1	156
2	19	4	30	21	29	20	26	19	15	8	191
3	23	2	21	10	33	9	22	14	6	9	149
BALF double strains	16	0	15	4	36	11	13	6	1	0	102
1	6	0	1	0	12	5	4	3	0	0	31
2	5	0	10	3	10	4	5	2	1	0	40
3	5	0	4	1	14	2	4	1	0	0	31
BALF triple strains	1	0	1	0	1	2	1	0	0	0	6
1	0	0	0	0	0	0	0	0	0	0	0
2	1	0	1	0	1	2	1	0	0	0	6
3	0	0	0	0	0	0	0	0	0	0	0

Where applicable the prevalence data are followed by the number of positive samples (nPCR)/total number of samples (nPCR) between brackets.

nPCR: nested polymerase chain reaction, BALF: bronchoalveolar lavage fluid, 1, 2, 3: respectively 1^st^, 2^nd^ and 3^rd^ batch of each herd.

#### Multiple locus variable number tandem repeat analysis (MLVA)

**Figure 1:**
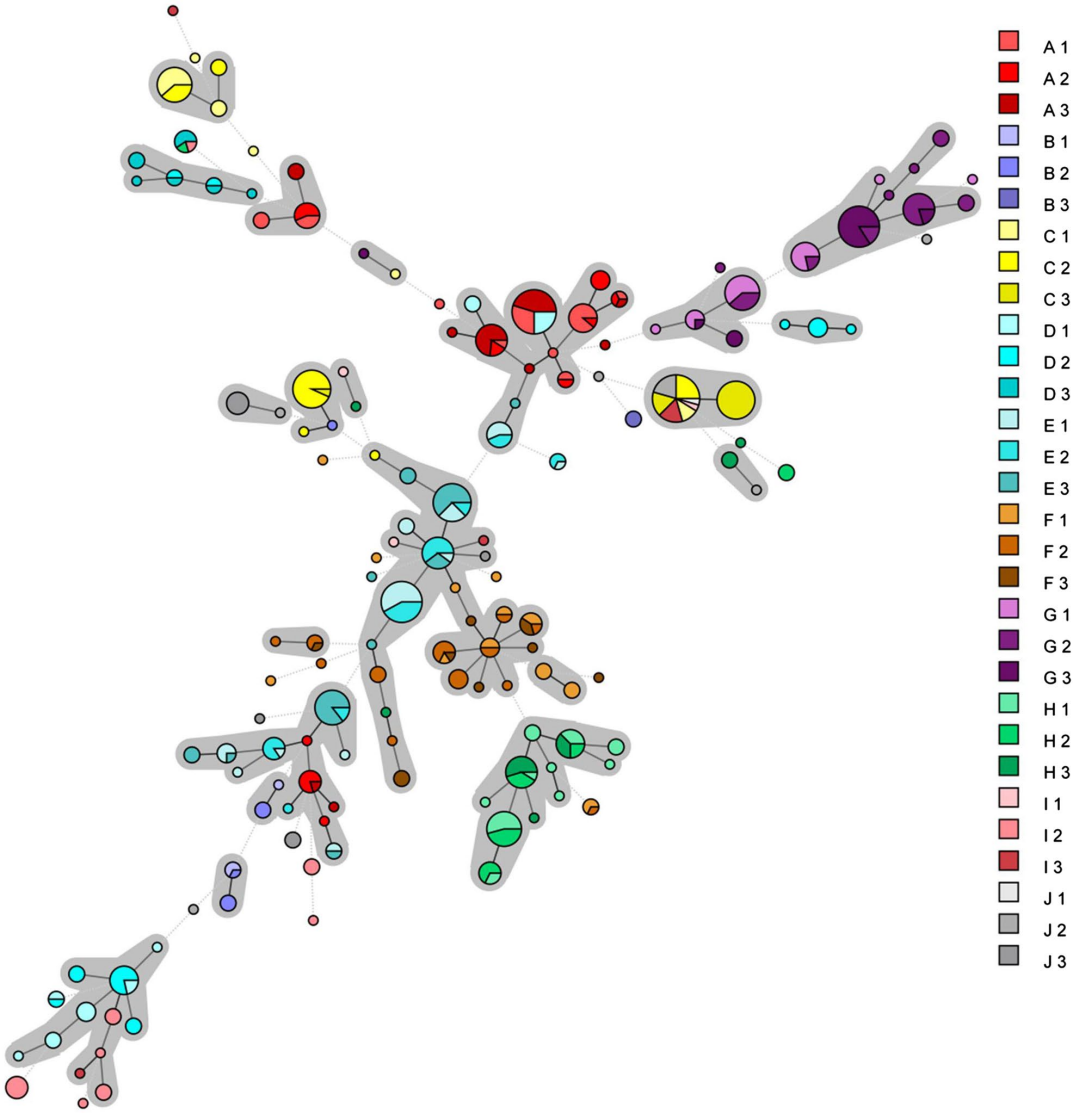
**Minimal spanning tree of all samples with a full MLVA profile in this study.** Samples in one colour belong to the same herd (A–J) and samples in a different shade of colour (1–3) belong to one of the three batches of each herd. Each circle represents a strain, the size of the circle is proportional with the number of samples belonging to a certain strain. Samples belonging to the same clonal complex, are marked with a grey background.

Samples that were positive using nPCR were submitted to MLVA. In the entire dataset, 135 different *M. hyopneumoniae* strains were found (Figure 1). The Hunter-Gaston discriminatory index for the complete dataset and for each herd separately is presented in Table 5.

**Table 5:** **The Hunter-Gaston discriminatory index was calculated for the complete dataset, for each VNTR, as well as for each of the 10 herds (A–J)**

	Hunter-Gaston discriminatory index									
Herd	A	B	C	D	E	F	G	H	I	J
	89.7	89.1	82.0	93.8	89.1	95.7	85.8	88.2	92.3	85.2
VNTR	h1	h5	p146	p97	Total					
	88.0	88.5	90.8	77.5	98.4					

h1, h5, p146, p97: four VNTRs in the genome of *M. hyopneumoniae* of which the length of the amplified fragments were measured.

Total: the Hunter-Gaston DI calculated for the entire dataset.

The average number of different strains per batch was 7 (min 1; max 13). The total number of strains and the number of different strains per batch are presented in Table 4.

The average number of different strains per batch in CAT 1, 2 and 3 were 1, 4 and 9, respectively (Table 3).

The most prominent strain was strain 2, with 24 detections in the whole data set of all herds. This strain was detected in herds C, I and J. Strain 113 was the second most prominent strain and was found in herd A and D for a total of 20 times. Strain 135 was only detected 5 times in the dataset, however in herd D, H and I. Hundred and ten strains out of 135 were only detected 5 times or less and 60 out of 135 strains were only detected once. Strain 2, 42, 45, 59, 61, 77, 78, 109 and 117 were found in each of the three sampling periods in herds C, F, F, E, E, H, H and A respectively. In herds B, D, G, I, J no strains were found circulating throughout all three sampling periods. In all herds, strains were identified that were detected in at least two out of three sampling periods, except for herd B. Most returning strains per batch were found in two consecutive sampling points. Five strains were found in the first and the third sampling point only: strain 2, 19, 21, 99 and 113 in respectively herd I, E, E, G and A. In a lot of lungs (102), two and a few lungs (6) three different strains were detected (Table 4). In herds B and J, no samples with two different strains were obtained. In two lungs of herd F, three different strains were found. In herds A, C, E, and G, always in the second batch, one sample with detection of three strains was found. In herds B, D, H, I and J no samples with three strains were detected.

#### Lung lesions

In total, 3820 lungs were evaluated at the slaughter line. The average (min.–max.) number of lungs scored per herd and per batch were 382 (200–494) and 127 (54–186), respectively. The average severity of *Mycoplasma*-like lesions in CAT 1, 2 and 3 were 0.78 ± 2.4%, 3.97 ± 10.7 and 5.54 ± 12.7. The average prevalence of pneumonia was 11.8, 23.2 and 29.7% in CAT 1, CAT 2 and CAT 3, respectively. The average prevalence of fissures was 29.2, 41.4 and 42.3% in CAT 1, CAT 2 and CAT 3, respectively. The average prevalence of pleurisy in CAT 1, CAT 2 and CAT 3 was 10.2, 21.2 and 29.1%, respectively (Table 3). The severity scores of *Mycoplasma*-like lesions, and the prevalence of pneumonia, fissures and pleurisy of each herd and each batch are shown in Table 6.

**Table 6:** **Descriptive results of lung lesions in the ten herds (A–J) and the three batches (1, 2, 3) within each herd:** severity of *Mycoplasma*-like lesions (*n* = 3605), prevalence of pneumoniae (*n* = 3605), fissures (*n* = 3605) and pleurisy (*n* = 3820)

Herd	A	B	C	D	E	F	G	H	I	J	Overall
*Mycoplasma*-like lesions	7.2 ± 12.6	0.6 ± 1.9	4.8 ± 11.5	1.8 ± 5.1	10.2 ± 17.3	2.7 ± 9.0	7.8 ± 14.4	3.9 ± 10.8	4.0 ± 11.6	2.3 ± 7.7	4.6 ± 11.6
1	7.3 ± 12.2	0.6 ± 1.9	1.7 ± 3.4	2.1 ± 4.2	5.9 ± 10.1	0.4 ± 1.4	6.4 ± 12.8	6.7 ± 13.8	2.1 ± 10.3	1.2 ± 2.9	3.6 ± 9.3
2	8.3 ± 12.7	0.6 ± 1.9	4.1 ± 9.2	1.3 ± 4.7	10.9 ± 17.9	0.9 ± 5.7	6.7 ± 14.0	2.1 ± 7.6	1.4 ± 6.0	0.1 ± 0.9	3.6 ± 10.5
3	6.3 ± 13.0	0.5 ± 1.9	8.6 ± 16.7	1.8 ± 5.9	13.2 ± 20.5	6.6 ± 13.3	10.4 ± 15.9	2.5 ± 9.1	7.9 ± 15.2	4.9 ± 11.5	6.4 ± 14.0
Pneumonia %	42 (83/197)	9 (29/310)	30 (69/229)	19 (81/432)	45 (205/455)	17 (70/422)	41 (179/433)	22 (91/407)	19 (71/382)	16 (54/338)	26 (932/3605)
1	41	10	22	26	42	7	38	37	11	17	26
2	48	10	30	13	45	4	35	16	7	2	20
3	38	8	38	17	48	37	51	12	35	28	32
Fissures %	46 (90/197)	32 (99/310)	34 (78/229)	38 (164/432)	48 (217/455)	37 (155/422)	39 (170/433)	52 (213/407)	51 (194/382)	32 (107/338)	41 (1487/3605)
1	29	31	50	48	28	22	31	34	38	25	33
2	33	33	17	32	54	30	44	66	46	33	41
3	71	32	42	34	59	57	42	57	63	34	49
Pleurisy %	15 (30/200)	8 (25/319)	29 (73/250)	35 (162/461)	37 (185/494)	27 (119/445)	16 (71/454)	33 (149/445)	29 (120/411)	2 (6/341)	25 (940/3820)
1	1	2	7	39	38	21	16	25	32	3	21
2	26	7	45	35	37	28	20	34	27	2	27
3	20	15	26	32	37	32	11	43	30	1	26

Where applicable the prevalence data are followed by the number of positive results (prevalence lung lesions)/total number of lungs scored (lung lesions) between brackets.

*Mycoplasma*-like lesions: minimum 0% and maximum 100% of the lung surface affected with pneumonia.

Number of lungs scored for severity of *Mycoplasma*-like lesions, prevalence of pneumonia and fissures: 3605 (because of severe pleurisy in some lungs it was not possible to evaluate all lungs entirely).

Number of lungs scored for prevalence of pleurisy: 3820.

SD: standard deviation, *n: * number, 1, 2, 3: respectively 1^st^, 2^nd^ and 3^rd^ batch of each herd.

#### Serology

The serological results for *M. hyopneumoniae*, PRRSv and H1N1, H1N2, H3N2 swine influenza viruses of each herd and each batch per herd are shown in Table 7.

**Table 7:** **Seroprevalence of** ***M. hyopneumoniae*** **, PRRSV, swine influenza subtypes H1N1, H1N2 and H3N2 in the ten herds (A–J)**

Herd	A	B	C	D	E	F	G	H	I	J	Overall
ELISA *M. hyopneumoniae n* = 600	85 (51/60)	33 (20/60)	90 (54/60)	77 (46/60)	100 (60/60)	98 (59/60)	97 (58/60)	87 (52/60)	20 (12/60)	48 (29/60)	74 (441/600)
ELISA PRRSv *n* = 240	100 (24/24)	96 (23/24)	96 (23/24)	100 (24/24)	100 (24/24)	100 (24/24)	100 (24/24)	100 (24/24)	100 (24/24)	100 (24/24)	99 (238/240)
HI influenza H1N1 *n* = 240	88 (21/54)	100 (24/24)	100 (24/24)	100 (24/24)	79 (19/24)	92 (22/24)	100 (24/24)	100 (24/24)	100 (24/24)	100 (24/24)	96 (230/240)
HI influenza H1N2 *n* = 240	96 (23/24)	100 (24/24)	100 (24/24)	100 (24/24)	100 (24/24)	100 (24/24)	100 (24/24)	100 (24/24)	100 (24/24)	100 (24/24)	100 (239/240)
HI influenza H3N2 *n* = 240	54 (13/24)	71 (17/24)	67 (16/24)	42 (10/24)	75 (18/24)	71 (17/24)	54 (13/24)	21 (5/24)	54 (13/24)	79 (19/24)	59 (141/240)

Seroprevalence data are followed with number of positive samples/total number of samples between brackets.

*n: * number, PRRSv: porcine reproductive, HI: hemagglutination inhibition titers, SIV: swine influenza virus.

### Associations between diversity of *M. hyopneumoniae* strains and lung lesions

The results of the final multivariable models are shown in Table 8. The severity of *Mycoplasma*-like lesions and the prevalence of pneumonia were higher in batches of CAT 2 than in batches of CAT 1 and significantly higher in batches of CAT 3 than in batches of CAT 1 (*P* = 0.064 for CAT 2 to CAT and *P* = 0.012 for CAT 3 to CAT 1 and OR: 1.30; *P* = 0.33 for CAT 2 to CAT 1 and OR: 2.08; *P* = 0.012 for CAT 3 to CAT 1, respectively for the severity of *Mycoplasma*-like lesions and the prevalence of pneumonia).

**Table 8:** **Results of the four final multivariable models, with severity of** ***Mycoplasma*** **-like lesions, prevalence of pneumonia, fissures and pleurisy as outcome variables**

	β	SE	OR	CI_min_	CI_max_	*P*
Severity of *Mycoplasma*-like lesions						
Intercept	−2.85	0.18	–	–	–	
CAT						0.027
CAT 2	0.35	0.19	–	–	–	0.064
CAT 3	0.51	0.20	–	–	–	0.012
Batch						<0.001
Batch 2	0.16	0.07	–	–	–	0.027
Batch 3	0.38	0.07	–	–	–	0.0021
Distance to public road^a^						0.0015
<100						
>100	0.24	0.08	–	–	–	0.0015
Stand empty farrowing unit^b^						<0.001
No						
Yes	−0.31	0.08	–	–	–	<0.001
Not always	0.03	0.09	–	–	–	0.70
Likelihood of pneumonia						
Intercept	−2.24	0.34	0.11	0.05	0.21	
CAT						<0.001
CAT 2	0.26	0.27	1.30	0.77	2.19	0.33
CAT 3	0.73	0.29	2.08	1.18	3.68	0.012
Batch						<0.001
Batch 2	−0.45	0.11	0.64	0.52	0.78	<0.001
Batch 3	0.42	0.10	1.53	1.25	1.86	<0.001
Number of herds surrounding the trial herd in a perimeter <5 km	0.01	0.00	1.01	1.01	1.01	<0.001
Vaccination gilts *M. hyopneumoniae* ^b^						<0.001
No						
Yes	−0.99	0.23	0.37	0.24	0.58	<0.001
Likelihood of fissures						
Intercept	−0.94	0.20	0.39	0.26	0.58	
CAT						0.008
CAT 2	0.30	0.18	1.35	0.95	1.93	0.094
CAT 3	0.53	0.20	1.70	1.15	2.50	0.007
Batch						<0.001
Batch 2	0.29	0.09	1.34	1.12	1.59	0.001
Batch 3	0.74	0.09	2.09	1.75	2.48	<0.001
Distance to public road^a^						0.009
<100						
>100	0.26	0.10	1.30	1.07	1.58	0.009
Stand empty farrowing unit^b^						<0.001
Yes	−0.40	0.10	0.67	0.55	0.82	<0.001
Not always	−0.76	0.14	0.47	0.36	0.61	< 0.001
Likelihood of pleurisy						
Intercept	0.25	0.72	1.29	0.32	5.25	
CAT						<0.001
CAT 2	−1.06	0.27	0.35	0.21	0.59	<0.001
CAT 3	−1.07	0.30	0.34	0.19	0.62	<0.001
Batch						0.002
Batch 2	0.35	0.10	1.41	1.16	1.72	<0.001
Batch 3	0.25	0.11	1.29	1.04	1.59	0.021
Cross fostering piglets during first week of life^b^						0.002
No						
<10%	−0.32	0.71	0.73	0.18	2.91	0.67
>10%	−1.96	0.77	0.14	0.03	0.64	0.040

Clinical signs *M. hyopneumoniae* with intensity cross fostering confounded. For severity of *Mycoplasma*-like lesions, a linear model was used. For the other outcome variables, a logistic model was used. For category (CAT), CAT 1 was the reference, for Batch, Batch 1 was the reference.

OR: odds ratio, CI: confidence interval, SE: standard error, *P: P* value, batch 1, 2, 3: referring to respectively the first, second and third sampling point in each herd, CAT (category) 1 (one *M. hyopneumoniae* strain per batch per herd), CAT 2: category 2 (two to six strains per batch per herd), CAT 3: category 3 (≥seven strains per batch per herd).

^a^<100 m is reference category.

^b^No is reference category.

In batches of CAT 2 and 3, there was a higher prevalence of fissures than in batches of CAT 1: CAT 2 to CAT 1: OR = 1.35; *P* = 0.094 and CAT 3 to CAT 1: OR = 1.70; *P* = 0.007).

Batches belonging to CAT 2 and 3 showed a lower prevalence of pleurisy (overall *P* < 0.001, CAT 2- CAT 1: OR = 0.35; *P* < 0.001 and CAT 3 to CAT 1: OR = 0.34; *P* < 0.001).

## Discussion

The present study revealed that, using MLVA, many different *M. hyopneumoniae* strains are present in slaughter pigs from different pig herds and batches within a herd. The results also showed that prevalence and severity of pneumonia lesions at slaughter were significantly higher in batches where more different *M. hyopneumoniae* strains were found.

The ten selected study herds can be considered as representative for other pig herds, as the housing, feeding and management practices are quite similar to most Belgian and West-European herds. Also the prevalence of lung lesions (pneumonia 26%, fissures 41%, and pleurisy 25%) was similar to the results of previous studies [21]. The fact that three different batches of pigs were investigated within a herd, allowed to account for possible variations over time within a herd.

The minimal spanning tree (MST) visualizes the phylogenetic relationship of the analysed strains. In comparison with previous work [12], the MST in the present study had a wide distribution, confirming the high diversity of the *M. hyopneumoniae* strains. A weighing factor was assigned to each locus according to its abundancy in the dataset. This allowed to take into account the importance of variation of less abundant loci. To the author’s knowledge this is the first time this approach is used for analysing the diversity of an organism. The Hunter-Gaston discriminatory index (98.4 when all four VNTRs are combined), confirmed that MLVA is a suitable and discriminatory technique to investigate genetic differences in *M. hyopneumoniae* [9]. The high variation in strains is also illustrated by the large number of different strains found at batch and even at animal level: in 102 pigs, two different strains were found, and in six pigs, three different strains were present. In theory more than three strains at animal level can be present and detected if multiple peaks in the electropherograms of each VNTR can be distinguished. In practice the MLVA-technique, has some limitations: the detection limit is 100 organisms/μL in bronchoalveolar lavage fluid and multiple strains can be detected if the differences in concentration are less than tenfold. Therefore, it cannot be excluded that only the dominant strains in the herd were detected [9]. Although it is known from previous studies that pigs may be infected with more than one strain [9, 11, 12], the results of the present study in vaccinated herds document a higher diversity of *M. hyopneumoniae* strains than shown by previous authors [6, 22–24]. The results also suggest that vaccination of piglets against *M. hyopneumoniae* does not lead to an important decrease in the diversity of *M. hyopneumoniae* strains in slaughter pigs. Some of the measures that might influence introduction of new strains in the farm might be purchasing and quarantine policy, swine density in the area, pig transport, all-in/all-out management and animal flow. It is not known whether contamination of the sampled pigs’ lungs had occurred through the scalding water. Marois et al. showed that although *M. hyopneumoniae* was detected in the scalding water, the lungs of SPF pigs remained negative by nested PCR [25].

The prevalence and severity of pneumonia lesions at slaughter were significantly higher in batches where more different *M. hyopneumoniae* strains were found, illustrating for the first time the importance of strain diversity at batch level. The severity of *Mycoplasma*-like lesions, the prevalence of pneumonia and the prevalence of fissures was significantly higher in batches of CAT 3 compared to CAT 1, and numeric differences were obtained when batches of CAT 2 were compared to CAT 1. The effect of batch was significant in all models, indicating that there is quite some variation between successive batches in a herd. It also indicates the importance of investigating more batches from each herd.

The exact explanation why more different *M. hyopneumoniae* strains at batch level may lead to more pneumonia lesions is not known. Some strains have been shown to be more virulent than others [26], and infection with a low virulent strain did not protect against subsequent infection with a highly virulent strain [13]. On the contrary, clinical symptoms and lesions were more severe in case of dual infection. It is therefore possible that also at batch level, the presence of many different *M. hyopneumoniae* strains may lead to more (severe) pneumonia lesions. Further research to explain the mechanisms is necessary. Charlebois et al. did not find a significant association between the number of different *M. hyopneumoniae* strains and severity of lung lesions in slaughter pigs [10].

To account for infection pressure possibly influencing the lung lesion data, rather than the number of different strains, all models were run with nPCR results included in the model. Only in the pneumonia model, the factor nPCR needed to be retained, but the overall conclusions for each model, including the pneumonia model remained the same (data not shown). Apart from *M. hyopneumoniae*, also other respiratory pathogens may be involved in pneumonia lesions [27]. Almost all pigs tested for swine influenza and PRRS virus were positive, and therefore, it is unlikely that these pathogens have biased the results. As lung lesions are multifactorial, the effect of potential non-infectious risk factors was taken into account in the multivariable models [14, 28, 29]. This allowed to investigate the effect of strain diversity in batches, apart from the effect of these risk factors. As the aim of the study was mainly to assess the importance of strain diversity, the other significant risk factors in the final models will only be discussed briefly.

The severity of *Mycoplasma*-like lesions was higher in batches from herds located further away from a public road (more than 100 versus less than 100 m), and when a stand-empty period in the farrowing unit was not practiced. The same two variables were also significant in the model for prevalence of fissures. One would expect that severity of lesions and prevalence of fissures to be higher in herds located closer to the public road, as this has been shown to be a risk factor for infection with *M. hyopneumoniae* [30]. One explanation could be that herds located further away from the public road are smaller herds with a lower biosecurity [31]. Also, all herds were located quite close to a public road in the present study. Not practicing a stand-empty period can be considered as one aspect of poor hygiene and biosecurity, which has been shown as a risk factor for respiratory disease [29].

The prevalence of pneumonia lesions was higher in case more other pig herds surrounded the herd, and when breeding gilts were not vaccinated against *M. hyopneumoniae*. Pig herd density in the region has been shown to be a risk factor for introduction of *M. hyopneumoniae* in the herd or for increased seroprevalence of *M. hyopneumoniae* [14, 32]. Purchasing gilts compared to no purchase was a risk factor for higher seroprevalence of *M. hyopneumoniae* in slaughter pigs [32]. Younger sows are more likely to transmit the infection to their piglets [33] and vaccination of breeding sows may lead to a lower infection level in weaned pigs [34] and to a lower prevalence of pneumonia in slaughter pigs [35].

Pleurisy was also measured in the study, as it is a common and economically important lesion. Experimental *M. hyopneumoniae* infection does however not lead to pleurisy lesions. Under field conditions, positive associations have been found between *M. hyopneumoniae* infection and pleurisy lesions [21], although the results are not consistent [36]. In the present study, although the descriptive values showed a higher prevalence of pleurisy when comparing CAT 2 and CAT 3 with CAT 1, the final models resulted in a higher number of different *M. hyopneumoniae* strains being associated with a lower prevalence of pleurisy, though the effect was small. A high intensity of mixing and cross-fostering pigs (>10%) compared to no cross-fostering of piglets was associated with a lower prevalence of pleurisy. This might be explained by the fact that cross-fostering may lead to a better colostrum intake by the piglets, resulting in better performance and health during their lifetime [37, 38].

MLVA testing on bronchoalveolar lavage fluid showed a high diversity of *M. hyopneumoniae* strains in slaughter pigs from herds vaccinated against *M. hyopneumoniae*. *Mycoplasma*-like lesions were more severe and the prevalence of pneumonia and fissures were higher when more different *M. hyopneumoniae* strains were present in a group of pigs. These results imply that inter- and intra-herd biosecurity measures decreasing the introduction of new *M. hyopneumoniae* strains, may lead to less (severe) pneumonia lesions in slaughter pigs.

## Abbreviations

*M. hyopneumoniae*: *Mycoplasma hyopneumoniae*
MLVA: multiple locus variable-number tandem repeat analysis
VNTRs: variable number of tandem repeats
PBS: phosphate buffered saline
nPCR: nested polymerase chain reaction
TBE: tris–borate–EDTA
OD: optical density
PRRSv: porcine reproductive and respiratory syndrome virus
SIV: swine influenza virus
CAT: category
OR: odds ratio

## References

[1] Thacker EL (2004). Diagnosis of *Mycoplasma hyopneumoniae*. J Swine Health Prod.

[2] Thacker E, Zimmerman JJ, D’Allaire S, Taylor DJ (2006). Mycoplasmal diseases. Diseases of swine.

[3] Minion FC, Lefkowitz EJ, Madsen ML, Cleary BJ, Swartzell SM, Mahairas GG (2004). The genome sequence of *Mycoplasma hyopneumoniae* strain 232, the agent of swine mycoplasmosis. J Bacteriol.

[4] Hutchison CA, Montague MG, Razin S, Herrmann R (2002). Mycoplasmas and the minimal genome concept. Molecular biology and pathogenicity of mycoplasmas.

[5] Madsen ML, Oneal MJ, Gardner SW, Strait EL, Nettleton D, Thacker EL, Minion FC (2007). Array-based genomic comparative hybridization analysis of field strains of *Mycoplasma hyopneumoniae*. J Bacteriol.

[6] Vranckx K, Haesebrouck F, Maes D, Pasmans F (2012) *Mycoplasma hyopneumoniae* diversity in pigs. Ph.D. Thesis, Ghent University, Department of Pathology, Bacteriology and Poultry Diseases, Faculty of Veterinary Medicine

[7] Torres-Cruz J, van der Woude MW (2003). Slipped-strand mispairing can function as a phase variation mechanism in *Escherichia coli*. J Bacteriol.

[8] Dos Santos LF, Sreevatsan S, Torremorell M, Moreira MAS, Sibila M, Pieters M (2015). Genotype distribution of *Mycoplasma hyopneumoniae* in swine herds from different geographical regions. Vet Microbiol.

[9] Vranckx K, Maes D, Calus D, Villarreal I, Pasmans F, Haesebrouck F (2011). Multiple locus variable number of tandem repeats analysis is a suitable tool for the differentiation of *Mycoplasma hyopneumoniae* strains without cultivation. J Clin Microbiol.

[10] Charlebois A, Marois-Créhan C, Hélie P, Gagnon CA, Gottschalk M, Archambault M (2014). Genetic diversity of *Mycoplasma hyopneumoniae* isolates of abattoir pigs. Vet Microbiol.

[11] Nathues H, Beilage EG, Lothar Kreienbrock L, Rosengarten R, Spergser J (2011). RAPD and VNTR analyses demonstrate genotypic heterogeneity of *Mycoplasma hyopneumoniae* isolates from pigs housed in a regionwith high pig density. Vet Microbiol.

[12] Vranckx K, Maes D, Del Pozo Sacristán R, Pasmans F, Haesebrouck F (2011). A longitudinal study of the diversity and dynamics of *Mycoplasma hyopneumoniae* infections in pig herds. Vet Microbiol.

[13] Villarreal I, Maes D, Meyns T, Gebruers F, Calus D, Pasmans F, Haesebrouck F (2009). Infection with a low virulent *Mycoplasma hyopneumoniae* isolate does not protect piglets against subsequent infection with a highly virulent *M. hyopneumoniae* isolate. Vaccine.

[14] Villarreal I (2010) Epidemiology of *Mycoplasma hyopneumoniae* infections and effect of control measures. Ph.D. Thesis, Ghent University, Department of Reproduction, Obstetrics and Herd Health, Faculty of Veterinary Medicine

[15] Morrison RB, Hilley HD, Leman AD (1985). Comparison of methods for assessing the prevalence and extent of pneumonia in market weight swine. Can Vet J.

[16] Kobish M, Blanchard B, Le Potier MF (1993). *Mycoplasma hyopneumoniae* infection in pigs: duration of the disease and resistance to reinfection. Vet Res.

[17] Michiels A, Piepers S, Ulens T, Van Ransbeeck N, Del Pozo Sacristán R, Sierens A, Haesebrouck F, Demeyer P, Maes D (2015). Impact of particulate matter and ammonia on average daily weight gain, mortality and lung lesions in pigs. Prev Vet Med.

[18] Stärk KDC, Nicolet J, Frey J (1998). Detection of *Mycoplasma hyopneumoniae* by air sampling with a nested PCR assay. Appl Environ Microbiol.

[19] Hunter PR, Gaston M (1988). Numerical index of the discriminatory ability of typing systems: an application of Simpson’s index of diversity. J Clin Microbiol.

[20] Rasbash J, Charlton C, Browne WJ, Healy M, Cameron B (2012) MLwiN Version 2.26. University of Bristol, Centre for Multilevel Modelling

[21] Meyns T, Van Steelant J, Rolly E, Dewulf J, Haesebrouck F, Maes D (2011). A cross-sectional study of risk factors associated with pulmonary lesions in pigs at slaughter. Vet J.

[22] Calus D (2010) Phenotypic characterization of *Mycoplasma hyopneumoniae* isolates of different virulence. Ph.D. Thesis, Ghent University, Departement of Pathology, Bacteriology and Poultry Diseases, Faculty of Veterinary Medicine

[23] Mayor D, Zeeh F, Frey J, Kuhnert P (2007). Diversity of *Mycoplasma hyopneumoniae* in pig farms revealed by direct molecular typing of clinical material. Vet Res.

[24] Nathues H (2011) Influence of *Mycoplasma hyopneumoniae* strain variation, environmental factors and co-infections on enzootic pneumonia in pigs. Ph.D. Thesis, University of Hannover, Field Station for Epidemiology, Bakum

[25] Marois C, Cariolet R, Morvan H, Kobisch M (2008). Transmission of pathogenic respiratory bacteria to specific pathogen free pigs at slaughter. Vet Microbiol.

[26] Vicca J, Stakenborg T, Maes D, Butaye P, Peeters J, de Kruif A, Haesebrouck F (2003). Evaluation of virulence of *Mycoplasma hyopneumoniae* field isolates. Vet Microbiol.

[27] Sibila M, Pieters M, Molitor T, Maes D, Haesebrouck F, Segalés J (2009). Current perspectives on the diagnosis and epidemiology of *Mycoplasma hyopneumoniae* infection. Vet J.

[28] Del Pozo Sacristán R (2014) Treatment and control of *Mycoplasma hyopneumoniae* infections. Ph.D. Thesis, Ghent University, Department of Reproduction, Obstetrics and Herd Health, Faculty of Veterinary Medicine

[29] Stärk KDC (2000). Epidemiological investigation of the influence of environmental risk factors on respiratory diseases in swine—a literature review. Vet J.

[30] Stärk KDC, Keller H, Eggenberger E (1992). Risk factors for the reinfection of specific pathogen-free pig breeding herds with enzootic pneumonia. Vet Rec.

[31] Amass F, Clark LK (1999). Biosecurity considerations for pork production units. Swine Health Prod.

[32] Maes D, Deluyker H, Verdonck M, Castryck F, Miry C, Vrijens B, de Kruif A (2000). Herd factors associated with the seroprevalences of four major respiratory pathogens in slaughter pigs from farrow-to-finish pig herds. Vet Res.

[33] Fano E, Pijoan C, Dee S, Torremorell M (2006). Assessment of the effect of sow parity on the prevalence of *Mycoplasma hyopneumoniae* in piglets at weaning IPVS.

[34] Ruiz AR, Utrera V, Pijoan C (2003). Effect of *Mycoplasma hyopneumoniae* sow vaccination on piglet colonization at weaning. J Swine Health Prod.

[35] Sibila M, Bernal R, Torrents D, Riera P, Llopart D, Calsamiglia M, Segalés J (2008). Effect of sow vaccination against *Mycoplasma hyopneumoniae* on sow and piglet colonization and seroconversion, and pig lung lesions at slaughter. Vet Microbiol.

[36] Fraile L, Alegre A, López-Jiménez R, Nofrarías M, Segalés J (2010). Risk factors associated with pleuritis and cranio-ventral pulmonary consolidation in slaughter-aged pigs. Vet J.

[37] Quesnel H (2011). Colostrum production by sows: variability of colostrum yield and immunoglobulin G concentrations. Animal.

[38] Declerck I, Dewulf J, Sarrazin S, Maes D (2016). Long-term effects of colostrum intake in piglet mortality and performance. J Anim Sci.

